# Efficacy and safety of curcumin and its combination with boswellic acid in osteoarthritis: a comparative, randomized, double-blind, placebo-controlled study

**DOI:** 10.1186/s12906-017-2062-z

**Published:** 2018-01-09

**Authors:** Armine Haroyan, Vahan Mukuchyan, Nana Mkrtchyan, Naira Minasyan, Srbuhi Gasparyan, Aida Sargsyan, Mikael Narimanyan, Areg Hovhannisyan

**Affiliations:** 1 “Erebuni” Medical Center, 14 Titogradian Street, 0087 Yerevan, Armenia; 2 Yerevan State Medical University of Armenia, Koryun 2, 0025 Yerevan, Armenia; 3 Anti-doping Service of Republican Centre of Sport Medicine, Acharyan Str., 2/6, Yerevan, Armenia

## Abstract

**Background:**

The aim of this clinical trial was to assess the efficacy and safety of curcuminoid complex extract from turmeric rhizome with turmeric volatile oil (CuraMed®) and its combination with boswellic acid extract from Indian frankincense root (Curamin®) vs placebo for the treatment of 40- to 70-year-old patients with osteoarthritis (OA).

**Methods:**

The effects of CuraMed® 500-mg capsules (333 mg curcuminoids) and Curamin® 500-mg capsules (350 mg curcuminoids and 150 mg boswellic acid) taken orally three times a day for 12 weeks in 201 patients was investigated in a three-arm, parallel-group, randomized, double-blinded, placebo-controlled trial. Primary outcome efficacy measures included OA physical function performance-based tests, the WOMAC recommended index of joint pain, morning stiffness, limitations of physical function, and the patients’ global assessment of disease severity.

**Results:**

Favorable effects of both preparations compared to placebo were observed after only 3 months of continuous treatment. A significant effect of Curamin® compared to placebo was observed both in physical performance tests and the WOMAC joint pain index, while superior efficacy of CuraMed vs placebo was observed only in physical performance tests. The effect size compared to placebo was comparable for both treatment groups but was superior in the Curamin® group. The treatments were well tolerated.

**Conclusions:**

Twelve-week use of curcumin complex or its combination with boswellic acid reduces pain-related symptoms in patients with OA. Curcumin in combination with boswellic acid is more effective. Combining *Curcuma longa* and *Boswellia serrata* extracts in Curamin® increases the efficacy of OA treatment presumably due to synergistic effects of curcumin and boswellic acid.

**Trial registration:**

This trial is registered at the database www.clinicaltrials.gov. https://clinicaltrials.gov/ct2/show/NCT02390349?term=EuroPharma&rank=1. Study registration number: NCT02390349.

## Background

Osteoarthritis (OA), a degenerative age-related disease that affects the joints, is the most common human musculoskeletal disorder and a leading cause of disability in elderly populations worldwide. The symptoms of OA include pain, morning stiffness, joint swelling, limited range of motion, decreased physical function, restriction of social activities and/or compromised work capacity. OA primarily affects articular cartilage and subchondral bone of synovial joints and results in joint failure, leading to pain with weight-bearing activities including walking and standing. Current OA treatments rely on analgesics, NSAIDs and cortisone, which manage pain and inflammation but have a wide range of adverse effects, drug interactions and contraindications and fail to restore the imbalances between catabolic and anabolic processes that underlie OA pathogenesis.

Curcumin (diferuloylmethane) is a bright yellow chemical derived from the turmeric (*Curcuma longa* L.) rhizome and has been reported to be a potent anti-inflammatory agent [1]. The clinical efficacy of curcumin in OA has been evaluated in many clinical trials [2–15]. Meta-analyses of eight random control trials (RCTs), with more than 800 participants with primarily knee OA, found scientific evidence that supports the efficacy of turmeric extract (about 1000 mg/day of curcumin) in treating OA [16, 17]. Curcumin may have some beneficial effects on knee pain and quality of life in patients with knee OA. Although curcumin is less effective at relieving pain than ibuprofen, it appears safe for short-term use and may reduce the need for rescue medication [17]. These studies failed to demonstrate a dramatic reduction in OA symptoms by curcumin but suggested strategies by which curcumin might be effective in OA. A challenge in curcumin research is its bioavailability. Due to its hydrophobic nature, curcumin has low absorption, fast metabolism, and fast systemic elimination [18, 19]. Hence, several studies have focused on improving curcumin bioavailability via different strategies, such as improving the solubility of curcumin using heat [20, 21], etheric oils [22–24], solubilizing polymers [25] or nanoparticles [26]; inhibiting glucuronidation of curcumin [27]; increasing absorption and decreasing systemic elimination by liposomal curcumin (Meriva® or SinaCurcumin®) [28, 29]. The bioavailability of curcuminoids can be enhanced by blending purified curcuminoids with turmeric volatile oil, which contains aromatic turmerone and various other sesquiterpenes as the main constituents of BSM-95 extract [30]. Thus, the results of ex vivo and pharmacokinetic studies of BCM-95 in animals and humans [22, 31] have indicated that the relative bioavailability of curcumin from BCM-95 complex is approximately 6.93-fold greater than that of normal curcumin and approximately 6.3-fold greater than that of liposomal curcumin-lecithin-piperine formula [22]. Pilot clinical studies evaluating BCM-95-containing supplements provide preliminary evidence of a beneficial effect for BCM-95 in rheumatoid arthritis [5], OA [32–34], and other conditions such as major depressive disorder, Alzheimer’s disease, hypercholesterinemia, oral submucous fibrosis and prostate cancer [31].

The gum-resin extract of *Boswellia serrata* Roxb. Ex Celebr. tree is used in Ayurvedic medicine for the treatment of asthma, rheumatisms, dysentery, skin ailments, ulcers, blood purification, etc. The anti-inflammatory and anti-arthritic activities of Boswellia are primarily attributed to boswellic acids [35–37]. The results of several randomized, placebo-controlled studies of various extracts from *B. serrata* suggest that they could be effective and safe alternative interventions for the management of OA [38–44].

Several systematic reviews have suggested the effectiveness and safety of curcumin- and boswellic acid-containing herbal products for treating OA [14–17, 37, 40, 45, 46]. However, the total number of RCTs included in the analysis, the total sample size, and the methodological and reporting quality of the primary studies were not sufficient to draw definitive conclusions. It was determined that more rigorous and larger studies are needed to confirm the therapeutic efficacy of turmeric for OA [16, 17, 46].

The chemical structures of curcumin and boswellic acid are quite different; consequently, their primary molecular targets are also likely different. The effectiveness of multi-target therapy and synergistic interactions between different biologically active molecules [47–49] gave rise to the idea to combine curcumin and boswellic acid in Curamin, a hypothesis that has been confirmed by the results o several studies [32–34]. The safety and efficacy of many formulations containing combinations of *Boswellia serrata* with other plant extracts (*Curcuma longa, Tinospora cordifolia, Zingiber officinale, Emblica officinalis, Withania somnifera)* have been evaluated in clinical studies of OA patients [50–52]. The formulations were found to be effective and safe, and no dose-related toxicity was found [32–34, 50–52]. Two studies also suggest significant benefits of both monodrug *CuraMed* (BCM-95) supplementation in humans [5] and its fixed combination with *Boswellia serrata* extract, Curamin [32–34]. However, since these studies have some limitations related to the small sample size and lack of a placebo group, more clinical studies and data on the clinical efficacy in a well-defined clinical condition are necessary.

The primary objective of this study was to compare the efficacy of extracts containing the combination of boswellic acid and curcumin (Curamin®) with curcumin (CuraMed®) or placebo in the treatment of degenerative joint disease (OA), and more specifically, to assess their effects in 40- to 77-year-old patients on the primary symptoms of OA such as joint pain, morning stiffness, and limitations of physical function. The secondary objective was to investigate the safety of CuraMed® and Curamin® treatment compared to placebo by assessing adverse events (AEs) during 12 weeks of repeated daily administration.

## Methods

### Participant eligibility

A phase II study of the efficacy of the Curamin® and CuraMed® supplements in patients with OA was carried out in Yerevan, Armenia, with the approval of the Health Research Ethics Board of the Yerevan Medical State University of Armenia. All participants signed written consent forms.

**Fig. 1:**
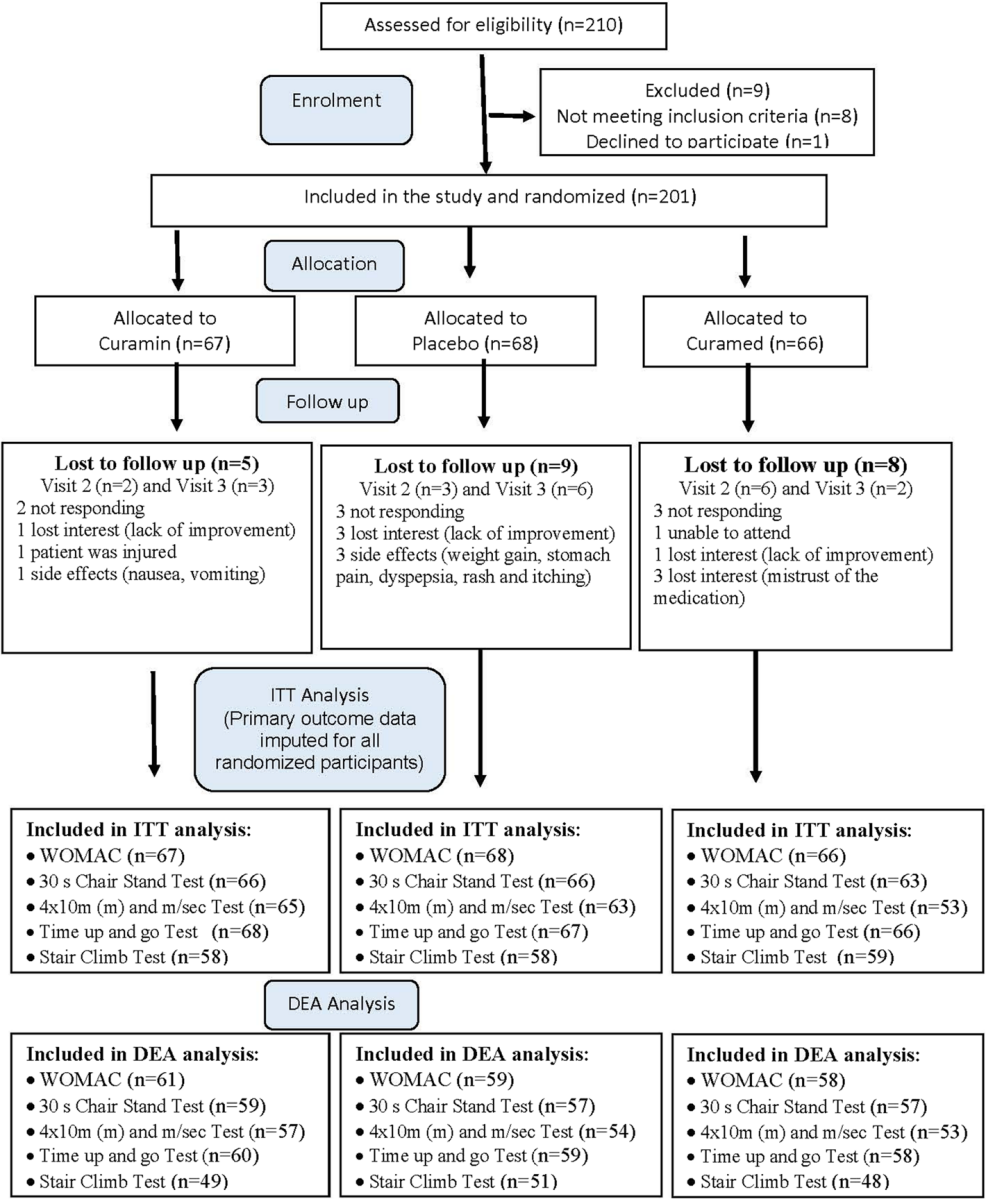
CONSORT Diagram - participant flow chart. ITT – intention to treat analysis, which includes all patients who completed the tests. DEA – dropouts excluded analysis, which includes only patients who completed all tests during all 3 visits

Two hundred ten males and females aged 40 to 77 years diagnosed with degenerative hypertrophic OA of knee bone joints were assessed for eligibility, and 201 patients were enrolled in the study and included in the intention to treat (ITT) analysis. All were randomized and allocated to three study interventions, of which 179 patients completed treatment, and 22 patients discontinued treatment during the study (Fig. 1). Participants were eligible for participation in this trial between September 2014, and May 2016. Individuals were recruited by doctors of “Erebuni” Medical Center and YSMU and among patients who visited the clinics.

### Selection of study population

During the initial visit to the study site, the inclusion and exclusion criteria were verified, and individuals interested in study participation received further information. Individuals, who met the criteria for study participation, made an appointment for medical screening. The patients who had taken a NSAID or turmeric were informed to stop for at least for 1 week (washout period) before the first visit to the doctors, when patients were randomized to study groups, passed physical performance measures (PPM) and Western Ontario and McMaster Universities Osteoarthritis (WOMAC) tests and provided blood for analysis.

#### Inclusion criteria

Patients diagnosed with degenerative hypertrophic OA (M 17, according to International Statistical Classification of Diseases and Related Health Problems 10th Revision (ICD-10) Version for 2014 of bone joints and verified by radiography (Grade I -III by Kellgren-Lawrence, 2007 radiographic grades [53]) were eligible. *Included* patients were those aged 40–80 years of either sex, body mass index (BMI) from 18 to 29 kg/m^2^, who provided written informed consent and who were capable of adequately participating in the study. There were 22 dropouts during the study (Fig. 1).

#### Exclusion criteria

Any of the following was regarded as a criterion for exclusion from the study:

- inflammatory or any secondary arthritis,
- moderate or severe synovitis (grades 2 and 3),
- tear of the meniscus,
- chronic diseases of the kidneys, liver, or gastrointestinal, cardiovascular, endocrine or nervous system,
- allergic anamnesis or drug intolerance,
- NSAIDs or analgesics used within 2 weeks prior to the study,
- glucosamine sulphate, chondroitin sulphate, intra-articular hyaluronate, or systemic or intra-articular glucocorticoids used within 3 month prior to the study,
- addiction to medicines, narcotics, or tobacco,
- pregnant or nursing.

### Study design

We designed a randomized, placebo-controlled, three-arm parallel-group, double-blind trial comparing the efficacy of Curamin with that of CuraMed and placebo (PL, negative control) (Fig. 1).

### Intervention and comparators

Curamin® 500 mg capsules and CuraMed® (BCM-95) 500 mg capsules were manufactured according to GMP and released by EuroPharma USA (Batch No. 141006 and 141,007).

Each capsule of CuraMed contains 552–578 mg of BCM-95® as a dry extract, (DER_native,_ 25:1) from *Curcuma longa* L. rhizome (extraction solvents: ethanol 99% *V*/V, ethylacetate. 100%) corresponding to 500 mg curcuminoids (curcumin −376 mg, demethoxycurcumin and bisdemethoxycurcumin – 124 mg) and 49–52 mg volatile oil from *Curcuma longa* L. rhizome containing to 22–23.4 mg aromatic turmerone [(6S)-2-methyl-6-(4-methylphenyl)-2-hepten-4-one]. Inactive excipients (120–149 mg) were phosphatidylcholine, medium chain triglycerides, glycerol, gelatine, and yellow beeswax (batch no. 141006).

One capsule of Curamin contains 350 mg BCM-95® and 150 mg *Boswellia serrata* Roxb. ex Colebr gum resin extract (DERnative, 10:1) consisting of 75% boswellic acids and 10% 3-O-acetyl-11-keto-boswellic acid (AKBA).

Placebo capsules contained 500 mg excipients, comprising a mixture of maltodextrin, calcium phosphate, gelatin, magnesium stearate, silica dioxide, FD&C yellow 5, FD&C yellow 6, and titanium dioxide. The appearance, smell and color of all three preparations were similar and organoleptically undistinguishable. Reference samples were retained and were stored at QC EuroPharma USA.

All herbal substances and herbal preparations were qualitatively tested by TLC and HPLC in accordance with specifications using appropriate reference standards. All analytical methods were validated for selectivity, accuracy and precision. All product samples were retained. CuraMed, Curamin and placebo were packed and labelled by EuroPharma USA per national requirements regarding their use for clinical trial investigations. The label also contained the drug name, study code and storage conditions.

#### Dosage

Participant received a labeled paper box containing either Curamin, CuraMed or placebo for a daily dose of one capsule (500 mg) orally, three times daily for 12 weeks. The capsules were provided in white plastic jars (252 capsules per jar) with a cap and sealed ring.

The Investigator was responsible for maintaining drug accountability records for the study products. Drug accountability for this study was carried out in accordance with standard procedures.

### Allocation and study procedures, follow up

#### Randomization and blinding

Treatment Randomization Code was generated prior the study and was provided to the Principal Investigator when all patients completed the treatment. It contained an information regarding the content and encoding of placebo, Curamin and CuraMed capsules. Containers were labelled in accordance with the Treatment Randomization Code generated using the random number generator in Microsoft Excel. That table contained three columns (A, B and C) filled with randomly distributed unique numbers from 1 to 210. Column A corresponded to Curamin, column B to placebo, and column C to CuraMed. Curamin, placebo and CuraMedd were assigned to containers for groups A, B and C, and the treatments were encoded by a qualified pharmacist (QP) for the course of the study medication randomization procedure at the manufacturing site.

#### Allocation concealment

The Treatment Randomization Code was kept by the QP at the investigational product manufacturing site (at sponsor) until the study was finalized.

#### Implementation

The allocation sequence was generated at the investigational product manufacturing site. Participants were enrolled by the Principal Investigator in collaboration with three study doctors. The patients were assigned to study groups A, B or C by the Principle Investigator, which were provided with the randomization code to the statistician at the end of the study. The study participants list, which identifying the patients and the study supplement packages (treatment numbers), was maintained by the Principal Investigator. This list was used for statistical analysis at the end of the study together with the *Treatment Randomization Code* received from QP. The treatment code providing the information about the actual assignments of groups A, B and placebo was revealed by the QP after statistical analysis of the results of study was completed and the data obtained from groups A, B and placebo were compared.

#### Blinding

Blinding for trial subjects was performed by using labeled jars containing capsules with an identical appearance. Study medication was delivered to the clinic pre-labeled and coded according to the randomization list. The randomization code was kept secret from the clinic and the participating investigators, and the code was only revealed after termination of the study. In this way, the investigators were also blinded to the study medication and placebo control, thus ensuring a double-blind design.

#### Evaluation of compliance

Individual subject compliance was ensured by recording the daily consumption of capsules. This was done using special forms provided to the subjects together with self-assessment questionnaires.

#### Follow up

During the 1st visit (day 0), study information and an informed consent were obtained. Upon study inclusion based on the results of medical examination of knee joint physical function, radiography and sonography, completing the Western Ontario and McMaster Universities Osteoarthritis (WOMAC) OA index assessment form and PPM tests, participants were randomly assigned to one of the three groups, provided a blood sample and received the test products (either CuraMed, Curamin, or placebo capsules), Table 1.

**Table 1:** Overall study design

Visit	Visit 1 Study site	At home	Visit 2 Study site	Visit 3 Study site
Study day	1	1–84	28	84
Information/Informed Consent	x			
Radiography	x			
Sonography				x
Blood sampling (ESR,CRP analysis)	x			x
PPM tests^a^	x		x	x
WOMAC test	x		x	x
Treatment		x		
Tablets intake count		x	x	x
Adverse Events		x	x	x

^a^PPM tests set includes 30-s Chair Stand Test (30s–CST), 40 m (4x10m) Fast Paced Walk Test (40 m FPWT), Timed Up and Go Test (TUG) and Stair Climb Test (SCT)

Participants completed WOMAC questionnaires, provided blood samples and underwent clinical examination by PPM tests on the 2nd (after 4 weeks, day 28) and 3rd (after 12 weeks) visits. Data regarding drug consumption was recorded independently by participants at home at baseline and every day throughout the 4 weeks of treatment.

During the 3rd visit, the radiography, sonography, PPM test, WOMAC index and compliance of study medication intake results were assessed by doctors.

CuraMed, Curamin, and the placebo were provided by the sponsor as equally-sized capsules that were identical in odor, taste and color. Two capsules of CuraMed, Curamin or placebo were orally administered to each participant after breakfast for 12 weeks. Packaging and labeling of the products were performed by EuroPharma USA.

The study sites were provided with randomly numbered packages of study medications (according to the randomization sequence generated at the production site), which were randomly assigned to patients.

After statistical evaluation of the data obtained in the study, the randomization code was disclosed to the statistician to assign each group of patients to placebo, CuraMed or Curamin treatment.

### Efficacy and safety evaluation

#### Primary outcomes

Efficacy primary outcome measures included:

- OA physical performance measures (PPM) using the OARSI recommended set of physical function performance-based tests including the 30-s chair stand test (30s–CST), 40 m (4 × 10 m) fast-paced walk test (40 m FPWT), the “timed up and go” test (TUG), the stair climb test (SCT) [54, 55].
- WOMAC recommended index of joint pain (five questions of the WOMAC questionnaire), morning stiffness (two questions), limitations of physical function (17 questions), and patients’ global assessment of disease severity considering the 48 h prior to the assessment (11 questions) [56].

The changes from baseline after 4 and 12 weeks of treatment were compared, and the significance of differences between the CuraMed group and the two control groups (Curamin and placebo) was estimated.

The OARSI set of performance-based tests of physical function consists of the following:

- 30s–CST, the maximum number of chair stand repetitions possible in a 30-s period;
- 40 m FPWT, a fast-paced walking test that is timed over 4 × 10 m for a total 40 m;
- TUG, the time (in seconds) taken to rise from a chair, walk 3 m, turn, walk back to the chair, and sit down while wearing regular footwear and using a walking aid if required;
- SCT, the time (in seconds) required to ascend and descend a flight of stairs. The number of stairs depends on individual environmental situations. Where possible, the 9-step stair test with a 20-cm step height and handrail is recommended.

#### Secondary outcomes

Secondary outcome measures included OA and inflammation sensitive hematological measures, including erythrocyte sedimentation rate (ESR) index, C-reactive protein (CRP), *and* CuraMed/Curamin-sensitive AEs.

CRP has been identified as a marker of chronic inflammation. Curcuminoids have been shown to lower circulating levels of CRP [57]. Both CRP and ESR were elevated in half of a cohort (*n* = 377) of patients with rheumatoid arthritis, systemic lupus erythematosus and OA [58].

AEs and intercurrent illness were given as examples of study events. The patients were told at the start of the study to immediately contact the investigator if intercurrent illness or any side effects developed.

### Sample size considerations

Assuming that the standardized difference in mean values between groups for the symptoms is 0.6 (this assumption is made on the results of previous studies where target differences and SD were estimated), and a power of 95% is acceptable to detect this difference as statistically significant at the 5% level, an estimated sample size of 180 patients (60 patients in each of the three groups) was calculated using monograms comparing sample sizes and power for three treatment groups in clinical trials and using Stat-Mate, version 2.00, 2004; GraphPad software. A total sample size of *N* = 180 is necessary to determine a significant interaction. As non-compliance is common in clinical trials, we prepared for a significant drop-out rate and increased our intended sample size to 210 participants.

### Statistical analysis

The data at each visit were recorded using a standardized assessment and transferred to an Excel database that was used for further data management. Statistical analyses were performed using GraphPad (San Diego, CA, USA) Prism software (version 3.03 for Windows); GraphPad Prism was also used to create supplemental graphs. The primary analysis followed ITT principles. All statistical tests were evaluated against a 0.05 level of significance and were two-sided tests. Before comparison of the data within or between groups, all data were checked and passed a normality test (* = 0.05).

The statistical analysis involved evaluating the patient’s change in scores from the initial visit (baseline) to the intermediate and final visits and at each scheduled visit of the study. The analyses were performed using “Observed” data.

Statistical evaluation of baseline characteristics was also performed on the 201 patients included in the trial. All data were checked for normality. Depending on the results of the normality test, the comparative assessment of the baseline characteristics between groups was made using:

- Kruskal-Wallis (KW) non-parametric one-way ANOVA rank-order test with post hoc Dunn’s multiple comparison test, or
- parametric one-way independent measures ANOVA with Dunnett’s multiple comparison test.

Analyses of changes within treatment groups during the study (repeated measures, before versus after) were performed using:

- paired t-tests (parametric data in two conditions, variables with normal distributions), or/and
- Wilcoxon signed rank test (nonparametric data in two conditions), or
- Friedman test for three repeated measures (nonparametric data), or
- one-way independent measures ANOVA (parametric data for three repeated measures).

The efficacy of the study supplements was assessed by comparing the mean changes from baseline (differences before and after treatment of every single patient) for each group using:

- Kruskal-Wallis (KW) non-parametric one-way ANOVA rank-order test with post hoc Dunn’s multiple comparison test, and/or
- parametric one-way independent measures ANOVA with Dunnett’s multiple comparison test (variables with normal distributions).

The statistical significance was set to an alpha of 0.05. The data were analyzed after all data collection.

## Results

### Study participants/disposition of participants and baseline variables

Between September 2014, and May 2016, 210 individuals were enrolled in the clinical trial. Of these, 201 (95.7%) participants met the inclusion criteria and were then randomized to either placebo or verum groups (Curamin, *n* = 67; CuraMed, *n* = 66). Among participants who were randomized, 22 (10.5%) were lost to follow-up due to various reasons, and the details are provided in Fig. 1 (CONSORT diagram). The ITT analysis included primary outcome data imputed for all randomized participants, while the dropout-free analysis included data of 149–178 patients who had completed/fulfilled all tests of physical performance during all visits. None of the participants withdrew from the study. The summary of the procedures performed by the participants is outlined in the flowchart (Fig. 1).

There were no significant differences in demographic and other measured characteristics between treatment and placebo groups at baseline. Table 2 shows the baseline demographics and clinical characteristics for responders who entered the randomization phase. The mean age of the participants was 56.2 years (range, 40 to 77) with a female predominance (approximately 93%) and an average BMI of approximately 29 kg/m^2^ (range, 18 to 49) at the time of enrolment. At the time of randomization, all participant characteristics were well balanced. The three groups did not exhibit any differences in demography at the beginning of the study (Table 2).

**Table 2:** Baseline characteristics of study participants allocated to interventions, *n* = 201

Treatment group	Curamin mean ± SD number of patients normality test *p* value	Placebo mean ± SD number of patients normality test *p* value	Curamed mean ± SD number of patients normality test *p* value	Intergroup comparison, mean difference^a^or difference in rank sum^b^ and *p* values	
Variables				Placebo vs Curamin	Placebo vs Curamed
Age (years)	57.91 ± 9.02 *n* = 67 *p* > 0.05	56.04 ± 8.55 n = 68 *p* > 0.05	54.65 ± 8.84 *n* = 66 *p* > 0.05	-1.87^a^ *p* > 0.05	1.39^a^ *p* > 0.05
Sex					
• Men	5 (7.5%)	3 (4.4%)	6 (9.1%)		
• Women	62 (92.5%)	65 (96.6%)	60 (90.9%)		
Body mass index (kg/m^2^)	29.81 ± 3.97 *n* = 67 *p* < 0.0001	28.81 ± 3.36 *n* = 68 *p* < 0.05	28.33 ± 3.6 *n* = 66 *p* > 0.05	18.9^b^ *p* > 0.05	3.8^b^ *p* > 0.05
WOMAC Osteoarthritis Index (WOI)	33.06 ± 15.56 *n* = 67 *p* > 0.05	33.37 ± 15.21 *n* = 68 *p* > 0.05	28.94 ± 13.20 *p* > 0.05	-2.69^a^ *p* > 0.05	1.43^a^ *p* > 0.05
WOMAC Joint pain index	6.39 ± 3.47 *n* = 67 *p* > 0.05	5.85 ± 3.25 *p* > 0.05	5.91 ± 2.77 *p* > 0.05	-0.53^a^ *p* > 0.05	-0.06^a^ *p* > 0.05
WOMAC morning stiffness index	1.91 ± 1.23 *n* = 67 *p* > 0.05	2.09 ± 1.29 *p* > 0.05	1.98 ± 1.29 *p* > 0.05	0.18^a^ *p* > 0.05	0.10^a^ *p* > 0.05
WOMAC limitation of physical function index	23.40 ± 11.30 *n* = 67 *p* > 0.05	8.50 ± 11.46 *p* > 0.05	8.20 ± 9.91 *p* > 0.05	-2.21^a^ *p* > 0.05	1.36^a^ *p* > 0.05
Physical performance test 1 30 s. Chair Stand Test Score	7.23 ± 3.45 *n* = 66 *p* > 0.05	33.37 ± 3.76 *n* = 66 *p* > 0.05	28.94 ± 3.71 *n* = 65 *p* > 0.05	1.27^a^ *p* > 0.05	0.30^a^ *p* > 0.05
Physical performance test 2 40 m Fast Paced Walk Distance Test (m - distance).	29.77 ± 6.16 *n* = 65 *p* > 0.05	27.58 ± 5.69 *n* = 63 *p* > 0.05	27.42 ± 5.80 *n* = 63 *p* > 0.05	-2.19^a^ *p* > 0.05	0.15^a^ *p* > 0.05
Physical performance test 3 40 m (4x10m) Fast Paced Walk Speed Test (m/s - speed).	1.39 ± 0.26 *n* = 65 *p* > 0.05	1.50 ± 0.30 *n* = 63 *p* > 0.05	1.50 ± 0.29 *n* = 63 *p* > 0.05	0.19^a^ *p* > 0.05	0.001^a^ *p* > 0.05
Physical performance test 4 Time up and go test (sec).	10.61 ± 3.14 *n* = 68 *p* > 0.05	9.44 ± 3.05 *n* = 67 *p* > 0.05	9.84 ± 3.37 *n* = 66 *p* > 0.05	-1.17^a^ *p* > 0.05	-1.84^b^ *p* > 0.05
Physical performance test 5 Stair Climb Test (sec).	14.24 ± 4.60 *n* = 58 *p* > 0.05	13.02 ± 4.28 *n* = 58 *p* > 0.05	13.03 ± 4.44 *n* = 59 *p* > 0.05	1.22^a^ *p* > 0.05	1.21^a^ *p* > 0.05
ESR, mm/h	9.550 ± 0.40 *n* = 60 *p* > 0.05	8.241 ± 0.38 *n* = 58 *p* > 0.05	8.089 ± 0.40 *n* = 56 *p* > 0.05	−0.007 to 2.44 *P* > 0.05 ns	−1.12 to 1.33 *P* > 0.05 ns
CRP, mg/L	2.918 ± 0.131 *n* = 60 *p* > 0.05	2.524 ± 0.11 *n* = 58 *p* > 0.05	2.609 ± 0.10 *n* = 56 *p* > 0.05	−0.022 to 0.73 *p* > 0.05^ns^	−0.46 to 0.30 *p* > 0.05^ns^

^a^One-way analysis of variance with post Dunnett’s Multiple Comparison Test

^b^Non-parametric Kruskal-Wallis test with post Dunn’s Multiple Comparison Test

### Efficacy of treatment

The primary outcome measures were pain and pain-related symptoms, such as difficulty of physical function of the knees, stiffness and poor physical performance measures. Secondary outcomes were hematological measures. The overall treatment effect defined as the effect size (ES) in terms of mean change from baseline in units of SD was calculated for each group (Table 3).

**Table 3:** Mean change from baseline (week 0) and endpoint (week 12) of primary outcome measures in three groups of patients (mean ± SD), mean difference between groups and effect size (ES, *d*_*Cohen*_*, g*_*Hedges*_) for mean changes from baseline of groups vs placebo group (95% CI), http://www.psychometrica.de/effect_size.html#anova

Study outcomes	Curamin	Placebo	Curamed	Intergroup comparison, mean difference or difference in rank sum$ and *p* values	
				Placebo vs Curamin	Placebo vs Curamed
WOMAC osteoarthritis total Index	7.38 ± 10.02*	2.26 ± 10.39	6.34 ± 11.38	**−5.12**	**−4.08**
				**−24.84** ^**$**^	**−17.18** ^**$**^
ES	0.50		0.37	***P*** < 0.05*	***P*** **> 0.05**
WOMAC joint pain index	2.02 ± 2.93*	0.69 ± 2.70	1.86 ± 2.95	**−1.32**	**−1.167**
				***P*** < 0.05*	***P*** **> 0.05**
ES	0.47		0.41		
WOMAC morning stiffness index	0.46 ± 1.35	0.14 ± 1.58	0.40 ± 1.54	**−12.43** ^**$**^	**−9.12** ^**$**^
				***P*** **> 0.05**	***P*** **> 0.05**
ES	0.22		0.17		
WOMAC limitation of physical function index	4.61 ± 6.66	1.34 ± 7.01	3.83 ± 7.56	**−3.27**	**−2.49**
				***P*** **> 0.05**	***P*** **> 0.05**
ES	0.48		0.34		
Chair Stand Test Score	1.74 ± 2.18*	0.44 ± 2.91	1.87 ± 2.41**	**1.30**	**1.44**
				***P*** < 0.05*	***P*** < 0.01**
ES	0.51		0.53		
Fast Paced Walk Test Speed, m/s.	0.10 ± 0.18**	0.01 ± 0.22	0.08 ± 0.05*	**0.12**	**0.10**
				***P*** < 0.01**	***P*** < 0.05*
ES	0.45		0.44		
Time up and go test, sec.	1.56 ± 2.04**	0.17 ± 0.84	0.78 ± 1.98	**−1.39**	**−0.59**
				***P*** < 0.01**	***P*** **> 0.05**
ES	0.89		0.40		
Stair Climb Test, sec.	2.03 ± 3.60**	0.22 ± 2.84	1.66 ± 2.37	**−1.82**	**−0.95**
				***P*** < 0.01**	***P*** **> 0.05**
ES	0.56		0.55		
Blood test 1: ESR	−2.75 ± 0.77	−4.98 ± 0.85	−4.12 ± 0.88	16.63^$^ *p* > 0.05	−7.09^$^ *p* > 0.05
Blood test 2: CRP	−0.787 ± 0.33	−1.376 ± 0.31	−1.274 ± 0.35	18.28^$^ *p* > 0.05	−8.67^$^ *p* > 0.05

*p < 0.05, ** - *p* < 0.01 vs placebo

$ - Non-parametric Kruskal-Wallis test with post Dunn's Multiple Comparison Test

The overall change from baseline in Curamin, Curamed the placebo group. It was estimated as the effect size (the standard mean difference between baseline and endpoint) and this was compared with the obtained from placebo group control. An increase in all domains represents improvement in symptoms

#### Western Ontario and McMaster universities osteoarthritis (WOMAC) index (WOI)

##### WOMAC total score

The total WOMAC index significantly decreased in all groups after 4 weeks of treatment (visit 2) and gradually decreased in the Curamin and CuraMed groups, while the effect was insignificant in the placebo group at the end of the study (visit 3, week 12) (Table 4). The improvements in the CuraMed and Curamin groups were 3.6- and 2.7-fold greater than that in the placebo group with corresponding ESs of 0.515 (*p* < 0.001) and 0.414 (*p* < 0.001) vs 0.146 (*p* = 0.154) (*p* < 0.0001***).

**Table 4:** Within (columns) and between (lines) group comparisons of WOMAC Total score (%)

	Curamin mean ± SD	Placebo mean ± SD	Curamed mean ± SD	Intergroup comparison, mean difference or difference in rank sum$ and *p* values	
				Placebo vs Curamin	Placebo vs Curamed
Visit 1 Baseline^a^	33.06 ± 15.56 *N* = 67	30.37 ± 15.21 *N* = 68	28.94 ± 13.20 *N* = 66	−2.69 *p* > 0.05	1.43 *p* > 0.05
Visit 2^a^	27.91 ± 16.2 *N* = 63	26.89 ± 13.9 *N* = 65	24.34 ± 14.44 *N* = 60	−1.02 *p* > 0.05	2.55 *p* > 0.05
Visit 3^a^	26.49 ± 17.0 *N* = 61	28.13 ± 15.57 *N* = 59	21.86 ± 14.36 *N* = 58	1.64 *p* > 0.05	6.27 *p* > 0.05
Within group Comparison^b^	*P* < 0.0001^¥^ *N* = 61	*P* > 0.05 *N* = 59	*P* < 0.05 *N* = 58		
Effect Size^a^ *d*_*Cohen*_ *, g*_*Hedges*_	**−0.404**	**−0.146**	**--0.515**		
Confidence interval	−0.754 − −0.054	−0.495 − 0.204	−0.873 − −0.156		
Mean change from baseline to Visit 2^a^	5.65 ± 6.97*** N = 63	3.65 ± 7.04*** N = 65	4.31 ± 8.75*** N = 60	−2.00 *P* > 0.05	−0.65 *P* > 0.05
Mean change from baseline to Visit 3^a^	7.38 ± 10.02*** N = 61	2.26 ± 10.39 *N* = 59	6.34 ± 11.38*** N = 58	**−5.12** **−24.84** ^**$**^ ***P*** < 0.05*	**−4.08** **−17.18** ^**$**^ ***P*** **> 0.05**

Within group comparison:

^a^Intention to treat analysis of all patients - One sample matched-pair t-test or Wilcoxon non-parametric test, * - *p* < 0.05, ** - *p* < 0.01, *** - *p* < 0.001

^b^Patients completed all tests – repeated measures ANOVA or Friedman’s test^¥^

Between groups comparison: One-way independent- measures ANOVA with post hoc Dunnett’s Multiple Comparison Test or Kruskal-Wallis test with post hoc Dunns Test^$^,

Effect size for mean differences of groups with different sample size; http://www.psychometrica.de/effect_size.html#anova. Confidence Coefficient – 95%

Comparing the changes from baseline across groups demonstrated differences in the effects of the intervention compared to placebo. Significant differences were revealed between the Curamin and placebo groups (*p* < 0.05), while no significant improvement in the CuraMed group compared to the placebo group was found (*P* > 0.05) (Table 4).

##### Pain

A statistically significant pain relief effect was observed in all study groups (Table 5). Even in the placebo group, the pain index decreased significantly after 4 weeks (visit 2) of treatment (*p* < 0.01). Comparing the pain index among groups between the beginning (visit 1, week 0) and end (visit 3, week 12) of the study (change from baseline, week 0 – week 12) showed that the pain index significantly decreased in both treatment groups. The improvement was significant in the Curamin group (ES −0.519; *p* < 0.001***) and in the CuraMed group (ES −0.734; *p* < 0.001***). In the placebo group, the ES of the pain index subscale was −0.185 between the beginning and end of the study (*p* > 0.05).

**Table 5:** Within (columns) and between (lines) groups comparison of WOMAC Pain Index subscale

	Curamin mean ± SD	Placebo mean ± SD	Curamed mean ± SD	Intergroup comparison, mean difference or difference in rank sum$ and *p* values	
				Placebo vs Curamin	Placebo vs Curamed
Visit 1 Baseline^a^	6.39 ± 3.47 *N* = 67	5.85 ± 3.25 *N* = 68	5.91 ± 2.77 *N* = 66	−0.53 *P* > 0.05	−0.06 *P* > 0.05
Visit 2^a^	5.02 ± 3.46 *N* = 63	4.92 ± 3.09 *N* = 65	4.37 ± 2.88 *N* = 60	−0.08^$^ *P* > 0.05	8.252^$^ *P* > 0.05
Visit 3^a^	4.49 ± 3.86 *N* = 61	5.22 ± 3.58 *N* = 59	3.84 ± 2.88 *N* = 58	0.73 *P* > 0.05	1.38 *P* > 0.05
Within group Comparison^b^	*P* < 0.0001 *N* = 61	*P* < 0.003 *N* = 59	*P* < 0.0001 *N* = 58		
Effect Size^a^ *d*_*Cohen*_ *, g*_*Hedges*_	**−0.519**	**−0.185**	**−0.734**		
Confidence interval	−0.872 − −0.166	−0.534 − 0.165	−1.098 − −0.369		
Mean change from baseline to Visit 2^a^	1.44 ± 1.98*** N = 63	0.89 ± 0.55** N = 65	1.47 ± 0.50*** N = 60	−0.55 *P* > 0.05	−0.02 −10.57^$^ *P* > 0.05
Mean change from baseline to Visit 3^a^	2.02 ± 2.93*** N = 61	0.69 ± 2.70 *N* = 59	1.86 ± 2.95*** N = 58	**−1.32** ***P*** **< 0.05**	**−1.167** ***P*** **> 0.05**

Within group comparison:

^a^Intention to treat analysis of all patients - One sample matched-pair t-test or Wilcoxon non-parametric test, * - *p* < 0.05, ** - *p* < 0.01, *** - *p* < 0.001

^b^Patients completed all tests – repeated measures ANOVA

Between groups comparison: One-way. Independent- measures ANOVA with post hoc Dunnett’s Multiple Comparison Test or Kruskal-Wallis test with post hoc Dunns Test^$^

Effect size for mean differences of groups with different sample size; http://www.psychometrica.de/effect_size.html#anova. Confidence Coefficient – 95%

Between-group comparisons of the changes from baseline demonstrated differences in the effect of the intervention compared to placebo. A statistically significant difference was found between the Curamin vs placebo groups (*p* < 0.05*) (Table 5).

##### Degree of difficulty of physical functions on knees and morning stiffness

Within-group comparison of the WOCAMP index and morning stiffness between the beginning (visit 1) and end (visit 3) of the study (change from baseline, visit 1 – visit 3) showed that the degree of difficulty to move the knees and stiffness significantly decreased in both treatment groups, while in the placebo group, this effect was significant only after 4 weeks of treatment but not at the end of the study (12 weeks, visit 3). However, between-group comparisons of the changes from baseline showed no significant difference between the effects of intervention compared to placebo (*p* > 0.05) (Table 3).

#### Clinical physical performance measures (PPMs)

##### Pain on standing from a chair

**Fig. 2:**
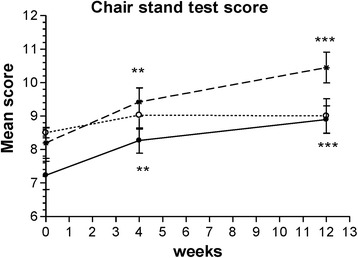
The changes with time in the maximum number of chair stand repetitions possible in a 30-s period at weeks 0, 4, and 12 in the Curamin, CuraMed and placebo treatment groups. Within-group improvements (*p < 0.05, **p < 0.01, ***p < 0.001) in physical performance tests at the end of the study (visit 3) compared to baseline (week 0)

The maximum number of chair stand repetitions possible in a 30-s period significantly from week 4 to week 12 of treatment only in the Curamin and CuraMed groups, Fig. 2 and Table 3). The ESs in the Curamin and CuraMed groups were 3.8- and 4.8-fold higher than in the placebo group, with corresponding ESs of 0.50 (confidence interval, 0.148–0.858) and 0.63 (confidence interval, 0.262–0.994) vs 0.13 (confidence interval, −0.222 - 0.481**)** (Table 3).

Between-group comparisons of the changes from baseline demonstrated differences in the effect of the intervention compared to placebo. Significant differences between the Curamin vs placebo groups (*p* < 0.05) and between the CuraMed vs placebo groups (*p* < 0.01) were observed (Table 3 and Fig. 2).

##### 40-m walking speed

**Fig. 3:**
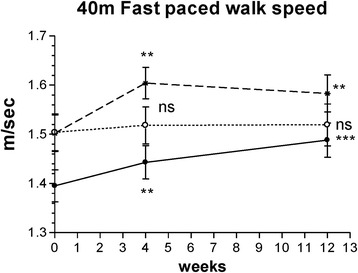
The changes with time in a fast-paced walking test timed over 4 × 10 m for a total 40 m at weeks 0, 4, and 12 in the Curamin, CuraMed and placebo treatment groups. Within-group improvements (*p < 0.05, **p < 0.01, ***p < 0.001) in physical performance tests at the end of the study (visit 3) compared to baseline (week 0)

The walking speed of a 40-m distance significantly increased from week 4 to week 12 of treatment only in the Curamin and CuraMed groups (Fig. 3 and Table 3). The ESs in the Curamin and CuraMed groups were 5.9- and 5.0-fold higher than in the placebo group, with an ESs of 0.38 (confidence interval, 0.022–0.733) and 0.32 (confidence interval, 0.042–0.679) vs 0.06 (confidence interval, −0.292 - 0.419**)** (Table 3).

Between-group comparisons of the changes from baseline demonstrated differences in the effect of the intervention compared to placebo. Statistically significant differences were found between the Curamin vs placebo groups (*p* < 0.01) and the Curamin vs placebo groups (*p* < 0.05) (Table 3 and Fig. 3).

##### Functional mobility by the TUG test

**Fig. 4:**
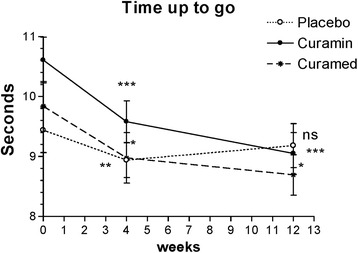
The changes with time in the time required to rise from a chair, walk 3 m, turn, and walk back to the chair at weeks 0, 4, and 12 in the Curamin, CuraMedand placebo treatment groups. Within-group improvements (*p < 0.05, **p < 0.01, ***p < 0.001) in physical performance tests at the end of the study (visit 3) compared to baseline (week 0)

The time taken to rise from a chair, walk 3 m, turn, and walk back to the chair significantly decreased at 4 weeks for all treatment groups. However, at the end of the study, the TUG time was significantly shorter only in the Curamin and CuraMed groups (Fig. 4 and Table 3). The ESs in the Curamin and CuraMed groups were 6.0- and 4.3-fold higher than in the placebo group, with ESs of 0.53 (confidence interval, −0.884 - -0.178) and 0.38 (confidence interval, −0.737 - -0.025) vs 0.09 (confidence interval, −0.439 - 0.262) (Table 3).

Between-group comparisons of the changes from baseline demonstrated differences in the effects of the intervention compared to placebo. A statistically significant difference was found between the Curamin vs placebo groups (*p* < 0.01), while no significant improvement in the CuraMed group compared to placebo was found (*P* > 0.05) (Table 3 and Fig. 4).

##### Pain on climbing stairs via the SCT

**Fig. 5:**
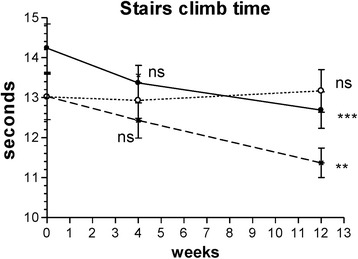
The changes with time in the time required to ascend and descend a flight of stairs at weeks 0, 4, and 12 in the Curamin, CuraMed and placebo treatment groups. Within-group improvements (*p < 0.05, **p < 0.01, ***p < 0.001) in physical performance tests at the end of the study (visit 3) compared to baseline (week 0)

The time required to ascend and descend a flight of stairs significantly decreased by week 12 only in the Curamin and CuraMed groups (Fig. 5 and Table 3). The ESs in the Curamin and CuraMed groups were 6.0- and 4.3-fold higher than in the placebo group, with ESs of 0.382 (confidence interval, −0.756 - -0.008) and 0.447 (confidence interval, -0.826 − -0.067) vs 0.036 (confidence interval, -0.333 − 0.405) in the placebo group (Table 3).

Blood tests to detect the effects of Cumarin® and Curamed® on chronic inflammation, the erythrocyte sedimentation rate (ESR) and C-reactive protein (CRP) showed no significant difference between the placebo group vs both treatment groups at baseline and the end of the study Table 6. Within group comparisons showed that their levels significantly increased at the end of the study in all groups, including the placebo group, but remained within the limits of 2–15 mm/h (ESR) and < 5 mg/L (CRP).

**Table 6:** Within (columns) and inter-groups (lines) comparisons of erythrocytes sedimentation rate (ESR) C-reactive protein (CRP) in blood of patients completed all testes

		Curamin *n* = 66 mean ± SD	Placebo *n* = 54 mean ± SD	Curamed *n* = 57 mean ± SD	Intergroup comparison, interval of confidence or difference in rank sum^$^ and *p* values	
					Curamin vs placebo	Curamed vs placebo
ESR, mm/h Reference range: 2–20 mm/h	Baseline Visit 1	9.55 ± 0.40	8.24 ± 0.38	8.09 ± 0.40	−0.007 to 2.44 *p* > 0.05	−1.12 to 1.33 *p* > 0.05
	Visit 3	12.30 ± 0.66**	13.22 ± 0.77**	12.21 ± 0.78**	−3.9 to 1.31 *p* > 0.05	−0.76 to 4.47 *p* > 0.05
	Change from baseline	−2.75 ± 0.77	−4.98 ± 0.85	−4.12 ± 0.88	16.63^$^ *p* > 0.05	−7.09^$^ *p* > 0.05
CRP, mg/L Reference range: < 5 mg/L	Baseline	2.92 ± 0.13	2.52 ± 0.11	2.61 ± 0.10	−0.022 to 0.73 p > 0.05	−0.46 to 0.30 p > 0.05
	Visit 3	3.70 ± 0.30*	3.90 ± 0.29**	3.88 ± 0.33***	−4.979^$^ *p* > 0.05	11.55^$^ *p* > 0.05
	Change from baseline	−0.787 ± 0.33	−1.376 ± 0.31	−1.274 ± 0.35	18.28^$^ *p* > 0.05	−8.67^$^ *p* > 0.05

** - *p* < 0.01 vs baseline, ***p < 0.001 vs baseline, * -*p* < 0.05 vs baseline, *p* > 0.05 - not significant

$ - Non-parametric Kruskal-Wallis test with post Dunn's Multiple Comparison Test

### Safety

The treatments were all well tolerated. In total, 13 AEs were observed in 13 of the 201 patients: 4 in the placebo group, 2 in the Curamin group and 7 in the CuraMed group, Table 7. Serious AEs were not observed. The patients who reported these minor events were distributed evenly throughout the Curamin group (3% of the sample size), the placebo group (5.9% of the sample size) and the CuraMed group (10.6% of the sample size). It is noteworthy that nausea was observed only in patients taking curcumin-containing supplementations. Serious AEs were not observed. Analysis of AEs observed in both the treatment and placebo groups revealed that the types and frequency of AEs were similar in all groups and were presumably not related to the treatment. All measured blood parameters remained within normal limits. There were no statistically significant differences between the groups at the end of the study.

**Table 7:** Adverse events: treatment emergent signs and symptoms (TESS) - those not seen at baseline. Number observed and rate with patient identification

Group	Number of patients	Number of AE/group,	Patient’s treatment code	Adverse events
Placebo	68	5.9%	10	Meteorism, gastro-esophageal reflux
			86	Weight gain after 28 days of treatment
			171	Stomach pain, dyspepsia, gastroesophageal reflux disease.
			202	Relapsing rash and itching at lower extremities which improved after medication withdrawal
Curamed	66	10.6%	2	Nausea for 2–3 days
			3	Meteorism, gastro-esophageal reflux, stomach pain
			34	Swelling of ankle joints
			105	Stomach pain, dyspepsia, gastroesophageal reflux for 5–6 days
			154	Bitter taste in mouth for a week
			185	Nausea, vomiting,
			186	Nausea for 2–3 days
Curamin	67	3.0%	175	Nausea, vomiting
			181	Nausea, heart beating

## Discussion

The strategy of drug discovery for the treatment of OA involves prevention of catabolic degradation and apoptosis of chondrocytes. These multistep processes involve many mediators of intra- and extracellular communication and several canonical pathways of intracellular signalling, including catabolic signalling pathways induced by the pro-inflammatory cytokines, interleukin-1β (IL-1β) and tumor necrosis factor-α (TNF-α), e.g. nuclear factor kappa-B (NF-kB)-mediated expression of the pro-inflammatory enzyme cyclooxygenase-2 (COX-2) [59]. The intervention that provides reduced pain, inflammation and/or stiffness associated with OA can help improve the joint mobility of patients with OA [60–62].

Results from clinical studies and numerous in vitro studies have indicated there are potentially beneficial effects of curcumin in treating chronic inflammation. Thus, many in vitro and animal studies demonstrated that curcumin acts as a master switch of inflammation by modulating several important molecular targets, including pro-inflammatory enzymes (COX and lipoxygenases [LOX]), transcription factors (e.g., NF-kB, AP − 1), cytokines (e.g., TNF, IL-1, IL-6, IL-18, and chemokines), kinases (Janus kinases), and other genes or enzymes [1, 63–66]. Curcumin was a potent inhibitor of the production of IL-1β-stimulated inflammatory and catabolic mediators (NO, PGE_2_, IL-6, IL-8, and MMP-3) by chondrocytes, suggesting that this natural compound could be efficient in treating OA [67]. The anti-osteoarthritic potential of curcumin has been widely studied in vitro, mainly in chondrocytes or on articular cartilage explants [66]. In vitro studies have shown that curcumin decreased the catabolic and degradation action of chondrocytes or cartilage explant models when stimulated with inflammatory IL-1β, lipopolysaccharides or TNF-α. Curcumin inhibited matrix degradation by decreasing the production of MMP-3, −9 and −13 via c-Jun-N-terminal kinases (JNK), NF-κB and the janus kinase-signal transducer and activator of transcription (JAK/STAT) pathway. Moreover, curcumin stimulated matrix synthesis by restoring type II collagen and glycosaminoglycan (GAG) synthesis [65]. In addition to its anti-catabolic effect, curcumin showed potent anti-inflammatory capabilities by inhibiting key inflammatory mediators (IL-6, IL-8, PGE2 and NO) and enzymes (COX-2 and iNOS) in both chondrocytes and cartilage explants. Curcumin also decreased chondrocyte apoptosis and antagonized inhibitors of cell growth and pro-apoptotic effects on synovial adherent cells. On the other hand, curcumin inhibited collagenase and stromelysin expression in both synoviocytes and chondrocytes [65]. However, it should be noted that detrimental toxic effects were associated with high doses of curcumin (50 μM) in a study of human OA chondrocytes [65].

Boswellia extract had anti-inflammatory effects on chondrocytes during in vitro and ex vivo experiments where serum from experimental animals was supplemented with Boswellia extract and co-cultured with human chondrocytes [68]. Boswellic acids significantly reduced the infiltration of leukocytes in the knee joint and, in turn, significantly reduced inflammation [69]. Several in vitro studies partially uncovered the molecular mechanisms underlying the anti-inflammatory properties of boswellic acid; these properties are associated with the prevention of collagen degradation and inhibition of pro-inflammatory mediators such as prostaglandins, COX, nitric oxide and NF-kB and down-regulation of the pro-inflammatory cascade [70, 71]. Boswellia extract also provided protection from IL-1β-induced death in human cultured chondrocytes and improved their glycosaminoglycan production. Boswellia extract also inhibited matrix metalloproteinase (MMP-3) production [68].

The results of several randomized, placebo controlled studies suggest extracts from *B. serrata* are effective and safe alternative interventions for the management of OA [38, 39, 41–43]. The benefits include controlling inflammatory responses by reducing pro-inflammatory modulators, and improving joint health by decreasing the enzymatic degradation of cartilage in OA patients [43]. A Cochrane review, with the purpose of assessing the benefits and harms of Boswellia in treating OA, concluded that *B. serrata* shows beneficial trends that warrant further investigation as the risk of AEs appears low [40]. It was uncertain if there was an increased risk of AEs or withdrawals with *B. serrata* extract due to the variable reporting of results across studies. The studies reported no serious AEs. Several formulations containing the combinations of *B. serrata* with other plants extracts were also evaluated in clinical studies for safety and efficacy in OA patients. The formulations were found to be effective and safe and no dose-related toxicity was found [32–34, 48–50].

A pilot, open label, comparative study evaluating a fixed combination of curcumin (BCM-95) and *B. serrata* extract (Rhulief™) vs Celecoxib in 28 patients with OA showed reduction of OA symptoms, such as pain relief, and improved physical performance (distance walked, range of motion) after 12 weeks of treatment [32–34]. It was equally effective as Celecoxib in alleviating crepitus and improving the range of joint movements. The authors concluded that the efficacy and tolerability of Rhulief™ were superior to those of Celecoxib for treating active OA. However, this conclusion was based on the results from an open label study with a small sample size, which was insufficient to draw definitive conclusions. More larger studies are needed to confirm the efficacy and safety of fixed combinations of curcumin (BCM-95) and *B. serrata* extracts for OA.

Another clinical trial of curcumin BCM-95 extract in RA patients showed that the curcumin group exhibited the highest percentage of improvement overall in the Disease Activity Score (DAS) and these scores were significantly better than those of patients in the diclofenac sodium group. Importantly, curcumin treatment was safe and not associated with any AEs [5]. However, the sample size of 15 patients in each group recruited in this RCT was too small, and the reporting quality was not in line with CONSORT recommendations. There was insufficient information regarding the random sequence generation, allocation concealment, blinding of participants and outcome assessment, the procedure for compliance, description of the study medication, monitoring, conducting the trial in accordance with ICH guidelines for GCP, and the voucher specimen (i.e., if sample was retained and, if so, where was it kept or deposited).

In this clinical trial, for the first time, we compared the efficacy and safety of turmeric rhizome extract BCM-95 (Curamed®) and its combination with Indian frankincense root (Curamin®) vs. placebo in Degenerative Joint Disease, specifically on the main symptoms of OA such as joint pain, morning stiffness, and the limitations of physical function in 40–77 year-old patients. Statistically significant pain relief effect was observed in all study groups (Figs. 2, 3, 4 and 5 and Tables 3 and 4). Even in the placebo group, the pain index decreased significantly after 4 weeks of treatment (*p* < 0.01). This observation is in line with other studies where placebo effects in OA were clearly demonstrated [72, 73]. Meta-analyses have indicated that more than 50% of study subjects respond positively to placebo treatment [72, 73]. Placebo is effective in the treatment of OA, especially for pain, stiffness and self-reported function. The size of this effect is influenced by the strength of the active treatment, the baseline disease severity, the route of delivery and the sample size of the study [72]. However, in our study, this effect decreased after 12 weeks of treatment (Figs. 2, 3, 4 and 5).

Intergroup comparison showed a significant difference between the Curamin and placebo groups after only 3 months of continuous treatment. Curamin significantly improved the physical functions of the patients and relieved pain compared to placebo according to the WOMAC index and all physical tests. Statistically significant differences between these groups of patients were observed for all objective physical performance tests and for the OA index (Table 3)**.** A beneficial effect of CuraMed compared to placebo was observed only in 2 of the 4 physical performance tests and in the WOMAC joint pain index. The ES was always less than in the Curamin group, suggesting that these plant extracts are more effective in combination. One possible explanation is that boswellic acid may increase the bioavailability of curcumin. In vitro, boswellic acid is known to reduce the level of β-glucuronidase in monosodium urate-activated neutrophyls [74]. Administration of boswellic acid to arthritic animals inhibited beta-glucuronidase activity in various sub-cellular fractions [75]. It is possible that the boswellic acid-induced decrease in expression of glucuronidase may reduce glucuronidation of curcumin leading to increased bioavailability and overall efficacy when curcumin is administered with boswellic acid in OA patients. However, to our knowledge, there is no published study demonstrating the effect of boswellic acid on the bioavailability of curcuminoids. Further pharmacokinetic and other studies are required to evaluate the interactions between curcumin and boswellic acid in vitro and in vivo.

The treatments were well tolerated. In total, 13 AEs (nausea, vomiting, stomach pain, gastroesophageal reflux and meteorism) were observed in 13 of 201 patients. The patients who reported these minor events were distributed evenly throughout the Curamin group (3% of sample size), the placebo group (5.9% of sample size) and the CuraMed group (10.6% of sample size). Major AEs were not observed.

## Conclusions

The results of this study showed that 12-week use of curcumin complex or its combination with boswellic acids reduces pain-related symptoms in patients with OA. Curcumin in combination with boswellic acid is more effective. Combining *Curcuma longa* and *Boswellia serrata* extracts in Curamin® increases the efficacy of treatment of OA presumably due to synergistic effects of curcumin and boswellic acid.

## Abbreviations

GMP: Good manufacturing practice
ICH: International council for harmonisation
WOMAC: Western Ontario and Mcmaster Universities Osteoarthritis Index
